# The Chinese, Their Present and Future

**Published:** 1892-05

**Authors:** 


THE CHINESE, THEIR PRESENT AND FUTURE: MEDICAL, POLITICAL, AND SOCIAL. By ROBERT COLTMAN, JR., M. D., Surgeon-in-Charge of the Presbyterian Hospital, at Teng Chow Fu; Consulting Physician of the American Southern Baptist Mission Society; Examiner in Surgery and Diseases of the Eye for the Shantung Medical Class; Consulting Physician to the English Baptist Mission, etc. Illustrated with fifteen fine photo-engravings. Philadelphia and London: F. A. Davis, publisher. 1891.

This book does not seem to be one that especially falls within the province of the medical practitioner or that belongs to the literature of medicine; but it is, nevertheless, an interesting portrayal of the methods and habits of the life of this strange race that inhabits the eastern world. In this book will be found a careful setting forth of the home-life of these singular people, and many anecdotes are told of them in an interesting and agreeable manner. The opium habit is touched upon, and much new light thrown upon this method of dissipation. The most interesting chapter to medical men is that upon leprosy, which deserves to be read by students of this horrible malady. An insight is given into the missionary life of the Celestial Empire, and a chapter is devoted to business opportunities, while in another the political situation of the Chinese is discussed. The book closes with a short chapter on future prospects of China, and it is handsomely illustrated with photo-gravures. Taken altogether, it is a most interesting and readable book.

